# The Effect of Exclusive Breastfeeding on Hospital Stay and Morbidity due to Various Diseases in Infants under 6 Months of Age: A Prospective Observational Study

**DOI:** 10.1155/2016/7647054

**Published:** 2016-04-17

**Authors:** Amarpreet Kaur, Karnail Singh, M. S. Pannu, Palwinder Singh, Neeraj Sehgal, Rupinderjeet Kaur

**Affiliations:** ^1^Department of Pediatrics, Guru Gobind Singh Medical College and Hospital, Faridkot 151203, India; ^2^Department of Pediatrics, Government Medical College and Hospital, Amritsar 143001, India; ^3^Civil Hospital, Tarn Taran 143301, India; ^4^Department of Medicine, Guru Gobind Singh Medical College and Hospital, Faridkot 151203, India

## Abstract

*Background*. Mother's milk is the best for the babies. Protective and preventive role of breast milk was evaluated in this study by assessing the relation of type of feeding and duration of hospital stay or morbidity.* Methods*. This prospective study was conducted in a tertiary care hospital and 232 infants in the age group of 14 weeks to 6 months formed the sample. There are two groups of infants, that is, one for breastfed and one for top fed infants. Statistical analysis was done and results were calculated up to 95% to 99% level of significance to find effect of feeding pattern on hospital stay due to various diseases and morbidity.* Results*. Prolonged hospital stay, that is, >7 days, was lesser in breastfed infants and results were statistically significant in case of gastroenteritis (*p* value < 0.001), bronchopneumonia (*p* value = 0.0012), bronchiolitis (*p* value = 0.005), otitis media (*p* value = 0.003), and skin diseases (*p* value = 0.047). Lesser morbidity was seen in breastfed infants with gastroenteritis (*p* value 0.0414), bronchopneumonia (*p* value 0.03705), bronchiolitis (*p* value 0.036706), meningitis (*p* value 0.043), and septicemia (*p* value 0.04).* Conclusions*. Breastfed infants have shorter hospital stay and lesser morbidity in regard to various diseases as compared to top fed infants.

## 1. Introduction

Since times immemorial breast milk has been considered as the best feed for infants. However in the last few decades research has been going on for finding a perfect formula as a substitute to human milk; still the truth remains that the human milk is the best for babies.

WHO has laid down guidelines of IYCF stressing the need of exclusive breastfeeding for 6 months which can be continued till 2 years of life [1]. These guidelines were changed in 2001 from exclusive breastfeeding of 4 months to 6 months highlighting defence of human milk against lower respiratory or gastrointestinal illnesses. Breast milk has IgA, oligosaccharides, lactoferrin, and other immune cells which provide bactericidal, virucidal, and fungicidal properties to protect infants during first year of life [2, 3].

WHO formed a global data bank on breastfeeding in 1991 which underwent several revisions and name was changed to global data bank on infant and young child feeding [4]. As per the data bank, 38% of the infants in the world are exclusively breastfed till 6 months of age and in India as per NFHS-3 only 27.6% of infants are breastfed till 4 to 5 months of age [5]. WHO has also fixed targets for 2025 to increase exclusive breastfeeding rate to 50% [6]. These guidelines are based on research of many years insisting on the importance of breastfeeding in preventing the morbidity and mortality because of infection.

These guidelines of WHO are supported by a retrospective cohort study of births in Scotland which included 502948 infants born between 1997 and 2007 and out of these 137905 (27%) infants were hospitalized at least once. It was observed that there was higher hazard ratio of infectious diseases in formula fed infants compared to breastfed infants till 6 months of age, that is, gastrointestinal infection (HR 1.59), upper respiratory tract infections (HR 1.28), lower respiratory tract infections (HR 1.5), urinary tract infection (HR 1.46), otitis media (HR 2.13), asthma (HR 2.06), allergies (HR 1.2), and fever (HR 1.13). No statistical significant relation was observed in cases of eczema. It was also observed that exclusively breastfed infants at 6 to 8 weeks had shorter length of hospital stay (2.81 days compared with formula fed infants 3.25 days) [7].

Similarly a large cluster-randomized trial, that is, Promotion of Breastfeeding Intervention Trial (PROBIT), was conducted between June 1996 and December 1997 with a 1-year follow-up in the Republic of Belarus in which total of 31 maternity hospitals and polyclinics participated. Sample population was divided into 2 groups, intervention group in which there was encouragement for initiating and maintaining breastfeeding and control group in which usual infant feeding practices were followed. It was observed that intervention group was more likely to exclusively breastfeed for 3, 6, 9, and 12 months and there was significant reduction in risk of one or more gastrointestinal tract infections (OR 0.6) and atopic eczema (OR 0.54) but no significant reduction in respiratory tract infection or otitis media was seen [8].

There have been various other studies showing protective and preventive effect of breastfeeding in cases of gastroenteritis [9–12], respiratory tract infection [10, 12–14], atopic dermatitis [15], asthma and allergies [15, 16], and acute otitis media [11, 13].

A study published in European Journal of Pediatrics highlighted that in addition to social and demographic factors like age of mother, education of mother, parity, mode of delivery, and anesthesia another important factor affecting breastfeeding establishment was practice followed by maternal grandmother and great-grandmother, as it is considered that breastfeeding behavior is controlled epigenetically and is transgenerational. Mothers and grandmothers who were breastfed were more likely to advise breastfeeding to their daughters and showed more chances of breastfeeding with odds ratio of 6.7 at 1 month and 3.71 at 3 months and the grandmothers who were breastfed chances of breastfeeding were again high with odds ratio of 4 at 1 month and 5.15 at 3 months. When both mother and grandmother were breastfed chance of breastfeeding was high with odds ratio of 26.98. The behavior was considered to be due to oxytocin receptors in hypothalamus and in medial preoptic area which are reduced in female offspring who received poor maternal care. No relationship was between breastfeeding outcome and type of paternal feeding [17].

Though there have been a lot of studies relating breastfeeding to morbidity, impact of breastfeeding in decreasing hospital stay has been studied to lesser extent.

We conducted this research to identify if any relation exists between feeding and hospital stay or morbidity.

## 2. Material and Methods

This was a prospective observational study conducted in Government Medical College and Hospital, Amritsar (India). All the infants in the age group of 14 weeks to 6 months admitted in the Government Medical College and Hospital, Amritsar (India), during 1.10.2007 to 30.09.2012 were taken into account as a basis for the study. The exclusion criteria were infants who expired due to serious illnesses, preterm infants, infants weighing less than 2.5 kg, and infants having APGAR less than 7. During this time period, 618 infants in age group of 14 weeks to 1 year were admitted and 27 infants were excluded on the basis of the exclusion criteria. Out of the remaining infants, 232 infants were in age group of 14 weeks to ≤6 months and they formed the sample of the study. A detailed pro forma was prepared and written consent obtained from the parents of the child.

Demographic data of the infants, mode of delivery, prelacteal feeds, type of milk, and previous illnesses were recorded. Anthropometric data was also recorded, that is, birth weight, length, and head circumference.

Morbidity was defined as the occurrence of gastrointestinal infections, lower respiratory infection, otitis media, meningitis, septicemia, urinary tract infection, skin infections, eye infections, congenital diseases, and inborn errors of metabolism.

Study was approved by institution's ethics committee. Socioeconomic status (SES) was calculated as per Kuppuswamy [18] scale.

Based on the feeding pattern infants were categorized as exclusively breastfed and top fed.

- *Exclusively Breastfed*. This comprised infants who were given only breastfeeding excluding water even.
- *Top Fed*. This included infants who were given milk other than exclusive breastfeeding, that is, cow's, buffalo's, goat's, and formula milk, both breast and other milk (mixed fed), and those who were given water in addition to breast milk.

All other details of feeding practices were taken as per pro forma. The nutritional status was determined using WHO growth charts for recording growth percentiles.

### 2.1. Clinical Assessment

Physical examination and necessary tests of each child were conducted. Detailed clinical examination and necessary investigations like Hb, TLC, DLC, and if needed blood C/s, urine C/s, chest X-ray, and so forth were also done.

### 2.2. Statistical Analysis

The number of infants was counted under different parameters and each relation was statistically tested through odds ratio and chi square test at 95% and 99% level of significance.

Analysis was done to find association between

- hospital stay (<7 days and >7 days) and feeding pattern in infants ≤6 months,
- mean hospital stay and feeding pattern in case of various diseases,
- morbidity in case of various diseases and feeding pattern in infants ≤6 months.

## 3. Results

The sample of the study was 232 infants ≤6 months of age. There were 142 (61.21%) males and 90 (38.79%) females exhibiting sex ratio of 1.58 : 1 (Table 1). The majority of the infants were from Class IV {105(45.26%)} of socioeconomic status as per Kuppuswamy scale while 49 (21.12%), 52 (22.41%), 18 (7.76%), and 8 (3.45%) belonged to Class V, Class III, Class II, and Class I, respectively (Table 1). Among the infants 51.72% were exclusively breastfed and 48.28% were top fed (Table 1).

In case of breastfed infants majority of infants suffering from gastroenteritis (94.12%), bronchopneumonia (88.24%), and bronchiolitis (100%) stayed for less than a week compared to top fed infants where majority of infants suffering from gastroenteritis (82.61%), bronchopneumonia (60.17%), and bronchiolitis (58.82%) stayed for more than a week (Table 2).

In meningitis/encephalitis and septicemia all the infants stayed in the hospital for more than a week because these diseases require prolonged treatment of 10 to 14 days or more.

In fever 85.17% of breastfed infants stayed for less than a week while in top fed infants 100% stayed for less than a week.

In otitis media and urinary tract infection, 100% of breastfed infants stayed for less than a week and 100% of top fed infants stayed for more than a week.

In skin diseases 100% of breastfed infants stayed for less than a week and 50% of top fed infants stayed for less than a week.

In case of infants in category of eye diseases 80% of breastfed infants stayed for less than a week and 100% of top fed infants stayed for more than a week.

Infants admitted with inborn errors of metabolism and surgical/orthopedics condition were only in breastfed category. In injuries/household accidents 100% of breastfed infants stayed for less than a week (Table 2).

The association between prolonged hospital stay (>7 days) of infants with feeding pattern was observed to be statistically significant in case of various diseases as the breastfed infants afflicted with gastroenteritis, bronchopneumonia, bronchiolitis, otitis media, or skin diseases had less prolonged hospital stay (Table 3). Mean hospital stay was less in case of breastfed infants afflicted with gastroenteritis, bronchopneumonia, bronchiolitis, otitis media, or skin diseases compared to top fed infants (Table 3).

No statistically significant association between feeding and hospital stay could be established in case of fever, urinary tract infections, injuries/accidents, birth defects, inborn errors of metabolism, surgical/orthopedic conditions, or eye diseases.

Out of the total 232 infants admitted in hospital 34.48% suffered from gastroenteritis, which shows that this is the most common disease among the infants. Out of these 34.48% infants, 14.66% were breastfed and 19.83% were top fed. It is observed that top fed infants are more prone to gastroenteritis and this may be so due to different constituents of animal milk or tinned milk and poor hygienic conditions of feeding bottle. Prevalence of bronchopneumonia is also much less being 7.33% in case of breastfed infants as compared to 12.07% in top fed infants. Also the number of infants admitted due to bronchopneumonia is less as compared to that of gastroenteritis. Bronchiolitis was seen in 10.78% of admitted infants and 3.45% were breastfed while 7.33% were top fed. Meningitis was seen in 3.02% of admitted infants and 0.43% were breastfed while 2.58% were top fed. There were 0.86% of breastfed infants diagnosed with septicemia and 3.45% of top fed infants diagnosed with septicemia. Fever was seen in 3.45% of admitted infants; 3.02% were breastfed while 0.43% were top fed. Otitis media was diagnosed in 3.45% of breastfed and 0.43% of top fed infants. Urinary tract infection was seen in 1.29% of breastfed and 0.43% of top fed infants (Table 4).

There is a statistically significant association between feeding pattern and morbidity among the breastfed infants of less than six months of age. Lesser morbidity in case of gastroenteritis, bronchopneumonia, bronchiolitis, meningitis, and septicemias was seen (Table 5).

There was no statistically significant association between breastfeeding and morbidity in case of fever, otitis media, urinary tract infection, injuries/household accidents, skin diseases, birth defects, inborn errors of metabolism, surgical/orthopedic conditions, or eye diseases.

## 4. Discussion

In our study statistically significant relation of shorter hospital stay in breastfed infants in comparison to top fed infants was found only in cases of gastroenteritis, bronchopneumonia, bronchiolitis, otitis media, and skin diseases (Table 3). No statistical significant relation was found in case of urinary tract infections and eye diseases.

In case of meningitis/septicemia all infants stayed for more than 7 days as treatment duration was 14 days.

Results almost similar to our study were shown by a study conducted by Cushing et al. in USA on 1202 healthy infants who were followed up for the first 6 months of life and incidence of lower respiratory infection in form of wheezing or cough or both was noted. It was shown that infants who were breastfed had shorter duration of hospital stay being 5 days as compared to 6 days in case of nonbreastfed infants (results were significant at 95% confidence interval) [19]. Another study from AIIMS, New Delhi, on admitted pneumonia infants also showed that lack of exclusive breastfeeding is associated with prolonged hospital stay, that is, >5 days, as 86% of infants who had prolonged hospital stay were not exclusively breastfed compared to 14% of exclusively breastfed [20].

Our study is supported by another study from Brazil on bronchiolitis patients in which it was concluded that exclusive breastfeeding was negatively correlated with hospital stay [21].

A study from Scotland concluded that exclusively breastfed infants had a shorter length of hospital stay (mean: 2.81 days) compared with formula fed infants (mean stay: 3.25 days) but length of hospital stay was not calculated for diseases individually [7].

We could not find any study relating to hospital stay with feeding pattern in cases of gastroenteritis, otitis media, urinary tract infections, or skin diseases separately.

In our study hospital admission because of gastroenteritis in breastfed infants was 14.66% while in top fed infants it was 19.83% (Table 4) which is in consonance with randomized trial from Belarus (PROBIT) in which there were 2 groups, intervention group in which breastfeeding was encouraged based on Baby Friendly Hospital Initiative and control group in which feeding pattern which was being followed was continued. In intervention group 3.2% of infants were admitted because of gastroenteritis while in control group there were 3.6% of infants, and odds ratio for gastroenteritis in intervention group was 0.92 (0.62–1.37) and in our study OR in breastfed infants with gastroenteritis was 0.5672 (0.3282–0.9804) and these results were statistically significant [8].

Our results were also supported by a study from Scotland in which hazard ratio for gastroenteritis in formula fed infants was 1.59 in comparison to 1.0 in case of exclusively breastfed infants [7].

A meta-analysis was published in 2011 on association of breastfeeding and diarrhea incidence, prevalence, hospitalization, and mortality. Eighteen studies were included in the analysis and higher relative risk of diarrhea was reported in predominantly breastfed (RR 1.26), partially breastfed (RR 1.68), and not breastfed (RR 2.65) infants in 0–5-month age group in comparison to exclusively breastfed infants [22].

In our study hospital admission because of bronchopneumonia in breastfed infants was 7.33% while in top fed infants it was 12.07% and in case of bronchiolitis admission in breastfed infants was 3.45% and in top fed infants it was 7.33% (Table 4) which is in consonance with randomized trial from Belarus (PROBIT) in which 17.9% of infants from intervention group (breastfed) were admitted because of respiratory tract infection (which included upper respiratory tract infection, otitis media, wheezing, croup, and pneumonia) while in control group 20.5% of infants were admitted and odds ratio for admission because of respiratory tract infection in intervention group was 0.85 (0.57–1.27) but these differences were small in intervention group compared to control group and they were not statistically significant while in our study OR was 0.4951 (0.2539–0.9657) and these results were statistically significant in case of bronchopneumonia and bronchiolitis (Table 5).

Similarly, study from Scotland also had hazard ratio of 1.50 for lower respiratory tract infections in formula fed infants compared to 1.0 in breastfed infants [7].

A meta-analysis on protective effect of breastfeeding in preventing hospitalization for respiratory illnesses also supports our study. Seven studies from developed countries were included and it was concluded that formula feeding is associated with a 3.6-fold increase in an infant's risk of respiratory hospitalization when compared with a minimum of 4 months of exclusive breastfeeding [23].

There was statistically significant association between breastfeeding and lesser hospitalization in our study (Table 5) in breastfed infants suffering from meningitis (OR 0.1485 (0.0176–1.2532)) and septicemia (OR 0.2203 (0.0458–1.061)) but any such association was not estimated in other studies.

In our study there was no statistically significant association between breastfeeding and morbidity in case of fever, otitis media, urinary tract infection, injuries/household accidents, skin diseases, birth defects, inborn errors of metabolism, surgical/orthopedic conditions, or eye diseases though in study from Scotland there was more morbidity in case of formula fed infants in cases of urinary tract infection (HR = 1.46), otitis media (HR = 2.13), and allergies (HR = 1.2) while there was lesser chance of eczema in cases of formula fed infants (HR = 0.73). In our study the reason for no statistically significant association between morbidity and urinary tract infections, otitis media, or allergies could be due to small number of infants admitted for these diseases [7].

In PROBIT study also there was reduction in risk of atopic eczema in intervention group (3.3% versus 6.3%; adjusted OR, 0.54; 95% CI, 0.31–0.95). The reason for no such association in our study could be because only hospitalized infants were the subjects and atopic eczema might have been treated on outpatient basis [8].

A review by Williams et al. included 6 studies from developed countries in which risk of hospitalization for diseases in breastfed was compared to nonbreastfed children. Two studies reported relative risk of hospitalization for any infection as 0.39 and 0.51 in breastfed compared to never breastfed infants. Four studies reported hospitalization for respiratory tract illnesses and 3 studies mentioned relative risk of hospitalization in breastfed compared to never breastfed infants as 0.6, 0.53, and 0.43 while one study reported that rate of hospitalization did not differ based on mode of feeding. Two studies reported hospitalization for gastrointestinal illnesses and relative risk of hospitalization in one of the studies was 0.54 in breastfed compared to never breastfed infants while another study found rates of hospitalization did not differ significantly based on mode of feeding [24].

A multicentre cohort study on risk of death and hospitalization in first half of infancy included infants from India, Ghana, and Peru and measured all-cause mortality, diarrhea specific mortality, mortality caused by acute lower respiratory infections, and hospital admissions were recorded and it was concluded that there were no significant differences in the risk of hospitalization between infants who were exclusively breastfed and those who were predominately breastfed or between those who were partially breastfed and those who were predominantly breastfed. But nonbreastfed infants were at a substantially higher risk of all-cause hospitalization (incidence rate ratio (IRR) = 3.39) and diarrhea specific hospitalization (IRR = 5.59) when compared with infants who had been predominantly breastfed [25].

## 5. Limitations of the Study

Our study had a limitation of being conducted in one region of India with 232 infants included in the study; however results were statistically and clinically relevant. Furthermore, we were not able to correct the hospital stay with other factors which might have influenced the length of stay of infants in the hospital like social factors.

## 6. Conclusions

Statistically significant association has been observed between exclusively breastfed infants and shorter hospital stay in case of gastroenteritis, bronchopneumonia, bronchiolitis, otitis media, and skin diseases in comparison with the top fed infants. Further it has been observed that morbidity rate of exclusively breastfed infants suffering from gastroenteritis, bronchopneumonia, bronchiolitis, meningitis, and septicemia is significantly less as compared to top fed infants.

## Figures and Tables

**Table 1:** Showing baseline data of infants.

	Number of infants	Percentage
Sex		
Male	142	61.21
Female	90	38.79
Socioeconomic status		
Class I	8	3.45
Class II	18	7.76
Class III	52	22.41
Class IV	105	45.26
Class V	49	21.12

	Age ≤ 6 months	
	Number	% age

Feeding pattern		
Exclusively breastfed	120	51.72
Top fed	112	48.28

**Table 2:** Showing number of *breastfed and top fed infants* in terms of *hospital stay* (<7 days and >7 days) due to various diseases.

Morbidity	Breastfed		Top fed	
	<wk	>wk	<wk	>wk
Gastroenteritis	32	2	8	38
Bronchopneumonia	15	2	11	17
Bronchiolitis	8	—	7	10
Meningitis/encephalitis	—	1	—	6
Septicemia	—	2	—	8
Fever	6	1	1	0
Otitis media	8	0	0	1
Urinary tract infection	3	0	0	1
Injuries/household accidents	8	0	0	0
Skin disease	7	0	1	1
Birth defects	4	1	1	0
IEM	2	2	0	0
Surgical/orthopedics	2	4	0	0
Eye diseases	8	2	0	1
Total	103	17	29	83

**Table 3:** Statistical analysis: hospital stay versus feeding pattern.

Morbidity	Odds ratio with 95% CI^#^	Chi square test (*χ* ^2^)^*∗*^	*p* value	Mean hospital stay (breastfed versus top fed)
Gastroenteritis	**0.0132** (0.0026–0.0664)	42.6	<0.05 and <0.001	2.59 days (1.10–6.28 days) versus 7.48 days (1.76–13.19 days).
Bronchopneumonia	**0.0863** (0.0164–0.4533)	10.39	0.0012	3.47 days (−0.34–7.41 days) versus 6.85 days (2.29–11.42 days).
Bronchiolitis	0	7.83	0.005	1.62 days (0.59–2.66 days) versus 5.47 days (−0.45–11.39 days).
Otitis media	0	9	0.003	2.62 days (1.14–4.11 days) versus 9 days.
Skin diseases	0	3.9	0.047	1.86 days (0.48–3.23 days) versus 6.0 days (−0.34–11.66 days).

^*∗*^Chi square test depicts absolute *χ* ^2^ value with *p* value. Significant positive association between lesser hospital stay and breastfeeding.

^#^OR (Odd's ratio) depicting chances of prolonged hospital stay in association with breast feeding against top feeding.

**Table 4:** Showing correlation of morbidity pattern and feeding pattern (breastfed versus top fed) in infants up to 6 months of age.

Morbidity	Breastfed		Top fed	
	Number	% age	Number	% age
Gastroenteritis	34	14.66	46	19.83
Bronchopneumonia	17	7.33	28	12.07
Bronchiolitis	8	3.45	17	7.33
Meningitis/encephalitis	1	0.43	6	2.58
Septicemia	2	0.86	8	3.45
Fever	7	3.02	1	0.43
Otitis media	8	3.45	1	0.43
Urinary tract infection	3	1.29	1	0.43
Injuries/household accidents	8	3.45	0	0
Skin disease	7	3.02	2	0.86
Birth defects	5	2.16	1	0.43
IEM	4	1.72	0	0
Surgical/orthopedics	6	2.58	0	0
Eye diseases	10	4.31	1	0.43
Total	120	51.72	112	48.28

**Table 5:** Tests of significance depicting morbidity pattern in relation to breastfeeding.

Morbidity	Odds ratio with 95% CI	Chi square test (*χ* ^2^)	*p* value
Gastroenteritis	0.5672 (0.3282–0.9804)	4.1601	0.0414
Bronchopneumonia	0.4951 (0.2539–0.9657)	4.3476	**0.03705**
Bronchiolitis	0.3992 (0.165–0.9659)	4.362	0.036706
Meningitis/encephalitis	0.1485 (0.0176–1.2532)	4.0743	0.043541
Septicemia	0.2203 (0.0458–1.061)	4.2218	0.0399
